# Perinatal relapse or recurrence rates in women reporting preconception anxiety and/or depression: a longitudinal study using linked data

**DOI:** 10.1007/s00737-025-01631-9

**Published:** 2025-10-14

**Authors:** Celia Rae, Lucy Leigh, Elizabeth Holliday, Catherine Chojenta

**Affiliations:** 1 School of Medicine and Public Health, University of Newcastle, Newcastle, NSW Australia; 2 Data Sciences, Hunter Medical Research Institute, Newcastle, NSW Australia

**Keywords:** Relapse, Recurrence, Perinatal, Anxiety, Depression

## Abstract

**Purpose:**

Few robust estimates of perinatal anxiety and/or depression in women who experienced anxiety and/or depression before pregnancy have been reported in the literature. This study calculated rates of perinatal anxiety and depression in women with a history of the disorders using data from the Australian Longitudinal Study on Women’s Health, the Australian Government’s Pharmaceutical Benefits Scheme and the Medicare Benefits Schedule.

**Methods:**

The analysis included two cohorts of Australian women. The first comprised 14,247 women born between 1973 and 1978 with nine waves of data collected from 1996 to 2018. The second cohort included 17,010 women born between 1989 and 1995 with data collected from six waves between 2013 and 2019. The proportion of women who reported anxiety and/or depression before having a child and who then reported anxiety and/or depression perinatally (i.e. relapse/recurrence) was calculated for first births and for any birth.

**Results:**

Compared to women who did not report preconception anxiety or depression, rates of perinatal anxiety and depression were higher among women reporting either condition pre-conceptually. For women in the 1973-78 cohort, the rate of perinatal anxiety was 24% (vs. 7%) and the rate of perinatal depression was 26% (vs. 10%). In the 1989-95 cohort, the rate of perinatal anxiety was 43% for women with preconception anxiety (vs. 18%) and the rate of perinatal depression was 41% for women with preconception depression (vs. 12%).

**Conclusions:**

Given the high rates of perinatal relapse or recurrence in women with preconception anxiety and/or depression, as well as the well-established risks to the health and development of their offspring, supporting these women to remain asymptomatic during the perinatal period is a priority.

## Article highlights

• Few estimates of perinatal anxiety/depression in women with preconception anxiety/depression (i.e. relapse/recurrence) have been reported.

• Data from over 30,000 women from two cohorts of the Australian Longitudinal Study of Women’s Health were analysed.

• Sensitivity analysis used data from the Australian Government’s Pharmaceutical Benefits Scheme and Medicare Benefits Schedule.

• Compared to women who did not report preconception anxiety/depression, women with a history of the illnesses experienced much higher rates of perinatal anxiety/depression.

• Given women with preconception anxiety/depression are at high risk of perinatal relapse/recurrence, supporting them to remain asymptomatic is a priority.

## Introduction

Anxiety and depression experienced perinatally (during pregnancy and the first year after birth) can significantly reduce maternal physical health and quality of life (Austin et al. 2017; Slomian et al. 2019), and impact the health and development of foetuses and infants, the consequences of which may extend into childhood and adolescence (Rogers et al. 2020). One large scale meta-analysis of over 195,000 mother-child dyads has demonstrated associations between perinatal anxiety and depression and poorer social-emotional, cognitive, language, motor and adaptive behavioural development of children (Rogers et al. 2020).

Epidemiological data indicate that nearly 12% of women experience perinatal depression worldwide (Woody et al. 2017). The prevalence of perinatal anxiety is estimated to be even higher at 21% (Fawcett et al. 2019). Moreover, comorbid anxiety and depression is estimated to affect from 4.6 to 47.6% of women during pregnancy (Thiagayson et al. 2013; González-Mesa et al. 2020) and from 7.1 to 13% in the year after birth (Falah-Hassani et al. 2016; Hou et al. 2023). In Australia, perinatal anxiety and/or depression has been estimated to affect 1 in 5 mothers, or 60,000 women every year (PwC Consulting 2019).

Associations between experiencing mental illness before pregnancy (i.e. preconception) and perinatal anxiety and/or depression have consistently been reported in the literature (Martini et al. 2015; Biaggi et al. 2016; Furtado et al. 2018; Hutchens and Kearney 2020; Míguez and Vázquez 2021). Not specific to the perinatal context, it has been hypothesised that depressive relapse (when symptoms return after a remission of the condition but before full recovery) or recurrence (a new episode after recovery) might be caused by mild dysphoria (unhappiness or dissatisfaction) activating dysfunctional attitudes that were formed during a person’s previous experience of depression, but supressed in remission (Segal et al. 2006). During the perinatal period, mild dysphoria could easily result from common challenges of pregnancy and early motherhood, including poor health in pregnancy, sleep deprivation, and pressures of meeting infant demands, often while caring for another child.

The causes of depressive relapse or recurrence during the perinatal period remain poorly understood. Among women with a history of depression, available evidence suggests recurrence risk is increased in pregnancy by anxiety and stress, partner dissatisfaction and conflict, low perceived support, personality disorders and problems with social or interpersonal functioning (Goodman and Tully 2009). Cessation of antidepressant medication has also been associated with increased depressive relapse in pregnancy (Cohen et al. 2006). Causes of relapse or recurrence of anxiety, particularly in the perinatal period, are relatively unknown. The limited evidence base suggests residual symptoms, personality disorders, extent of improvement from treatment, and stressful life events may be predictive of anxiety relapse and recurrence in general (Lorimer et al. 2021).

Few studies have reported robust estimates of perinatal anxiety and/or depression relapse or recurrence rates among women with preconception anxiety and/or depression. Of the available studies, data broadly indicate significantly higher perinatal anxiety and depression rates among women with a history of these disorders, compared to those with no history. In a German longitudinal study of 306 pregnant women, 197 of whom had experienced anxiety and/or depression before pregnancy, Martini et al. (2015) reported a recurrence rate of 20.8% for women with preconception depression, compared to 11.9% of the general population estimated to experience perinatal depression (Woody et al. 2017). While anxiety is estimated to affect 21% of all perinatal women, 56% of women with preconception anxiety experienced the condition again perinatally (Martini et al. 2015). For those who had experienced preconception comorbid anxiety and depression, the rate of recurrence was found to be 29.2% for perinatal depression and 63.1% for perinatal anxiety (Martini et al. 2015).

A prospective cohort study from the Netherlands measured rates of depressive relapse or recurrence from 12 weeks into pregnancy to 12 weeks after the birth and found 9.4%, or eight of 85 women, relapsed or developed a recurrence (Molenaar, et al.^ 2019^). No studies calculating relapse or recurrence rates for Australian women have been reported in the literature.

The present study aimed to describe the proportion of women with preconception anxiety and/or depression who experienced a recurrence of their condition in the perinatal period, and to compare the rates to the proportion of women without such a history who experienced perinatal anxiety and/or depression, using data from the Australian Longitudinal Study on Women’s Health (ALSWH), the Australian Government’s Pharmaceutical Benefits Scheme (PBS) and the Medicare Benefits Schedule (MBS).

While causation would not be proven by the analysis, if rates of perinatal anxiety and/or depression were higher for women with preconception anxiety and/or depression, the results could provide sufficient evidence to argue that investment in further research and even a pilot intervention is warranted and necessary. If women with preconception anxiety and/or depression experienced the same or lesser rates of perinatal anxiety and/or depression than women without a history of the disorders, the findings might indicate successful universal prevention programs are sufficient to support women with preconception anxiety and/or depression to remain asymptomatic.

## Materials and methods

### Recurrence analysis

ALSWH is a national, longitudinal, population-based study of the health and wellbeing of 57,000 Australian women occupying four distinct age cohorts at inception, extending for nearly 30 years. ALWSH is the largest study of its type in Australia and participants broadly represent the Australian female population. The ALSWH study aims to understand the social, behavioural and economic factors which influence the health and wellbeing of Australian women, and their use of health and related services.

Two ALSWH cohorts were included in this recurrence analysis. The first cohort of 14,247 women were born from 1973 to 1978 and were invited to participate in nine waves of data collection from 1996 to 2018. Their most recent survey demonstrated a retention rate of 57.3%. The second cohort was born from 1989 to 1995 and involved 17,010 women in six waves of data collection from 2013 to 2019. A retention rate of 55.9% was measured at their latest survey. Details are provided in Tables 1 and 2.

**Table 1:** Demographics of women in the 1973-78 ALSWH cohort at baseline

Variable		Total (*N* = 14,247)
Age at survey	Mean (SD)	24.59 (1.47)
Accessibility/Remoteness Index of Australia (ARIA+)	Major cities of Australia	4950 (51%)
	Inner regional Australia	2960 (31%)
	Outer regional Australia	1445 (15%)
	Remote Australia	224 (2.3%)
	Very Remote Australia	67 (0.7%)
	Missing (% of total sample)	4601 (32.3%)
Marital status	Married	2353 (24%)
	De facto	1999 (21%)
	Separated	108 (1.1%)
	Divorced	34 (0.4%)
	Widowed	4 (0.0%)
	Never married	5141 (53%)
	Missing (% of total sample)	4608 (32.3%)
Highest qualification	No formal qualifications	144 (1.5%)
	Year 10 or equivalent	907 (9.7%)
	Year 12 or equivalent	2251 (24%)
	Trade/apprenticeship	293 (3.1%)
	Certificate/diploma	1975 (21%)
	University degree	3207 (34%)
	Higher university degree	561 (6.0%)
	Missing (% of total sample)	4909 (34.5%)
Proficiency in speaking English	English-speaking at home	12,903 (91%)
	Very well	970 (6.9%)
	Well	194 (1.4%)
	Not well	61 (0.4%)
	Not at all	6 (0.0%)
	Missing (% of total sample)	113 (0.8%)
Hours spent in paid employment	1–15 h	891 (6.3%)
	16–24 h	871 (6.2%)
	25–34 h	911 (6.5%)
	35–40 h	3024 (22%)
	41–48 h	1317 (9.4%)
	49 h or more	402 (2.9%)
	Not in paid work	6643 (47%)
	Missing (% of total sample)	188 (1.3%)

**Table 2:** Demographics of women in the 1989-95 ALSWH cohort at baseline

Variable		Total (*N* = 17,010)
Age at survey	Mean (SD)	20.53 (1.69)
ARIA + Grouped	Major cities of Australia	12,627 (74%)
	Inner regional Australia	2893 (17%)
	Outer regional Australia	1127 (6.6%)
	Remote Australia	143 (0.8%)
	Very Remote Australia	48 (0.3%)
	Overseas	161 (0.9%)
	Missing (% of total sample)	11 (0.1%)
Current relationship status	I am single	6478 (38%)
	I am in a relationship (not living together)	5612 (33%)
	I am living with a partner	3308 (20%)
	I am engaged	791 (4.7%)
	I am married	504 (3.0%)
	I am divorced	6 (0.0%)
	I am separated	47 (0.3%)
	Other (please specify)	81 (0.5%)
	Missing (% of total sample)	183 (1.3%)
Highest level of education completed	Year 10 or below	572 (3.4%)
	Year 11 or equivalent	693 (4.1%)
	Year 12 or equivalent	7313 (43%)
	Certificate I/II	687 (4.1%)
	Certificate III/IV	2600 (15%)
	Advanced Diploma/Diploma	1128 (6.7%)
	Bachelor degree	3442 (20%)
	Graduate diploma/Graduate certificate	153 (0.9%)
	Postgraduate degree	239 (1.4%)
	Missing (% of total sample)	183 (1.3%)
Fluency in English	Yes	16,782 (99.7%)
	No	45 (0.3%)
	Missing (% of total sample)	183 (1.3%)
Hours spent per week in paid work	0	3704 (22%)
	1–15	4801 (29%)
	16–29	3350 (20%)
	30–34	1051 (6.2%)
	35–40	2804 (17%)
	41–49	838 (5.0%)
	50 or more	272 (1.6%)
	Missing (% of total sample)	190 (1.3%)

Based on available literature, it was anticipated that women with preconception anxiety and/or depression experienced higher rates of perinatal anxiety and/or depression than women without a history of the disorders. If this were proven to be the case, the results would have implications for current and future service delivery, therefore it was considered most appropriate to analyse the data from the most recent cohort of the ALSWH study (born 1989-95). However, in acknowledgement that the youngest participants of the cohort were only 24 years old and analysing recurrence rates over the reproductive life course of women with preconception anxiety and/or depression would minimise the risk of bias, it was decided to include the older cohort as well (born 1973-78).

Rates of perinatal anxiety and/or depression were also calculated amongst women who did not report experiencing anxiety or depression before pregnancy to allow for a meaningful comparison to the women who did. For example, rates of perinatal depression were calculated in each cohort for women who did not report experiencing depression before pregnancy.

In the ALWSH study, presence of anxiety and/or depression at each wave was established by participant self-report to questions such as, “Have you ever been diagnosed with or treated for: Depression?” or “Have you been diagnosed with or treated for the following in the last 12 months: Anxiety disorder?” An indicator was then created for a positive response, noting the earliest occasion the participant reported each condition.

Given anxiety and/or depression was assigned based on participant self-report (as opposed to an established measure or a clinical diagnosis), and more detailed questions were not asked, the type of anxiety or depressive disorder a participant experienced could not be determined. As such, the term ‘anxiety’ was used in this study to describe all anxiety disorders and ‘depression’ all depressive disorders listed in the DSM-V (APA 2013).

The ALSWH dataset further contains the birth dates and prematurity status for all children born to participants. An antenatal period was established for each birth of each woman, defined as 40 weeks for a full-term birth and 35 weeks for premature birth, which aligned with figures presented in the 2022 National Perinatal Data Collection preliminary update (AIHW 2022). For each birth, women were also asked about whether they experienced perinatal anxiety and/or depression.

As it was not possible to discern whether perinatal anxiety and/or depression represented a relapse or recurrence for individual participants from the ALSWH data, the term ‘recurrence’ has been used in this study to represent both terms. For the analysis, recurrence was defined as the number of women who experienced a preconception disorder (e.g. depression) who then experienced the condition again during the perinatal period (e.g. perinatal depression). To establish recurrence, preconception anxiety and/or depression was defined as the presence of anxiety and/or depression at any survey wave prior to the antenatal period. Perinatal anxiety and/or depression occurrence at an earlier birth also constituted preconception anxiety and/or depression for all subsequent births.

The proportion of women reporting anxiety and/or depression at the birth of their first child (i.e. recurrent anxiety and/or depression), and among any of their births, was calculated and reported alongside 95% confidence intervals. The proportion of women who did not report anxiety or depression before pregnancy, but who then developed perinatal anxiety and/or perinatal depression was also determined with 95% confidence intervals.

### Sensitivity analyses

There was risk of bias in the recurrence estimates due to under-identifying women with preconception anxiety or depression as a result of missing survey data or participants having children before the survey began. The impact of this potential bias on estimates of recurrent depression and recurrent anxiety was examined via two sensitivity analyses performed in each cohort.

The first sensitivity analysis aimed to identify women who were excluded from the denominator (due to having no reported history of anxiety and/or depression), regardless of whether they had missing surveys. The ALSWH datasets were linked with data from two schemes subsidised by the Australian Government; the PBS, which subsidises prescription medication, and the MBS, which subsidises professional medical services such as consultations, procedures and operations. A list of relevant antidepressant and anxiolytic medications was sourced from MIMS, an independent database of medicine information commonly used by Australian healthcare professionals, and these were matched with their corresponding Anatomical Therapeutic Chemical (ATC) PBS codes. MBS item numbers were sourced from the online MBS database and included psychological services from psychiatrists, general practitioners and allied health providers.

In recognition that some antidepressants and anxiolytics can be used to treat medical conditions unrelated to anxiety and depression, as well as that the MBS item numbers included in the analysis were for general psychological services not limited to any one diagnosis, depression or anxiety was only established if participants had relevant data from both the PBS and MBS. If a participant was both prescribed an antidepressant or anxiolytic, and a psychological service was claimed on their behalf by a relevant provider within one year, preconception anxiety or depression was assigned at whichever event occurred first. The PBS/MBS, wave history and perinatal indicators were then combined in the main analysis to assign a final history indicator noting the earliest report of anxiety and/or depression.

Approximately half of the relevant ATC codes identified were for medications to treat depression. The second half of codes were for medications that could be used to treat either anxiety or depression. To examine depressive recurrence, medicines described only as antidepressants in MIMS were included in the analysis to determine a lower bound on the number of additional women to include in the denominator. To establish an upper bound, all possible antidepressant and anxiolytic medications were included. While no medications were identified with the sole intention of treating anxiety, a similar upper bound analysis for anxiety was performed for each cohort by including all medications that treat both anxiety and depression.

A second sensitivity analysis was then performed for each cohort and condition by randomly imputing increasing proportions of the women who had been excluded from the denominator because they reported no anxiety or depression, but who also had missing survey data. This approach addresses the possibility that the missing survey data may not be missing completely at random (MCAR), and that women with anxiety or depression may have been more likely to have missing survey data. The proportions of recurrent depression and recurrent anxiety were then recalculated.

## Results

### 1973-78 Cohort Participants

Participants of the 1973-78 cohort were aged from 18 to 23 years at their baseline survey in 1996, and from 40 to 45 years at the most recent survey in 2018. Compared to data from the 1996 Australian Census, demographic characteristics of these women are similar to other Australian women of the same age; however somewhat more ALSWH participants were born in Australia and identified as non-Indigenous (Brown et al. 1999).

At baseline, 1332 ALSWH participants reported a history of depression (11.07%) compared to a national average of 10.7% in a similar age group in 1997 (AIHW 1999). For anxiety, 567 ALSWH women reported experiencing the condition in 1996 (4.71%), compared to 13.8% of Australian women of a similar age estimated to have experienced anxiety using the Composite International Diagnostic Interview (CIDI) in the 1997 National Survey of Mental Health and Wellbeing (ABS 1998); a difference that may reflect poor mental health literacy regarding anxiety as observed in other studies (Coles and Coleman 2010; Furnham and Lousley 2013; Hadjimina and Furnham 2017).

Of the 14,247 women included in the 1973-78 cohort, 2163 reported experiencing depression before a child in one of the nine surveys. Of the women who also provided information about their perinatal mental health, around a quarter (25.69%) reported a recurrence of their depression with their first child, and 23.03% reported a recurrence with any of their children. In comparison, 6,030 women did not report experiencing depression at any wave of the ALSWH study before pregnancy and 9.87% of these women subsequently reported perinatal depression with at least one of their children.

For those who reported preconception anxiety (*n* = 1224) and provided perinatal data, 23.79% experienced the disorder again with their first child and 23.04% experienced it with any of their children. In comparison, of the 6,522 women in the 1973-78 cohort who did not report a history of anxiety, 7.11% (*n* = 464) then experienced perinatal anxiety.

Of the 918 women who reported a history of both anxiety and depression and provided perinatal data for their first child, 28.12% experienced recurrent depression, 27.32% experienced recurrent anxiety and 18.78% experienced comorbid perinatal anxiety and depression. For any of their children, 25.52% of women experienced recurrent depression, 25.96% experienced recurrent anxiety and 17.68% experienced comorbid anxiety and depression.

Of the women who did not report a history of both anxiety and depression, 11.33% (*n* = 761) experienced perinatal depression, 7.37% (*n* = 495) experienced perinatal anxiety and 4.51% (*n* = 303) experienced comorbid perinatal anxiety and depression for any child.

Recurrence is defined as the number of women who experienced a preconception disorder (e.g. depression) who then experienced the condition again during the perinatal period (e.g. perinatal depression).

More details are provided in Tables 3 and 4.

**Table 3:** Recurrence in women from 1973-78 cohort with preconception anxiety or depression, alongside the comparison group with no such history

	Recurrence with first child			Recurrence with any child				Comparison group (at first child)				Hx vs. No Hx at first child
Preconception disorder	No. of women in analysis	No. of women with recurrence	% (95% CI)	No. of women in analysis	No. of women with recurrence	% (95% CI)	Preconception disorder	No. of women in analysis	No. of women with perinatal depression	No. of women with perinatal anxiety	% (95% CI)	Relative risk (95% CI)
Depression	1094	281	25.69 (23.1, 28.27)	2041	470	23.03 (21.20, 24.85)	**No depression**	6030	595	Not calculated	9.87 (9.11, 10.62)	2.60 (2.29, 2.95)
Anxiety	601	143	23.79 (20.39, 27.20)	1224	282	23.04 (20.68, 25.40)	**No anxiety**	6522	Not calculated	464	7.11 (6.50, 7.77)	3.34 (2.83, 3.96)

**Table 4:** Recurrence in women from 1973-78 cohort with a history of both anxiety and depression, alongside the comparison group with no such history

	Women with history							Women with no history (at first child)			Hx vs. No Hx at first child
	Recurrence with first child				Recurrence with any child						
Perinatal disorder	No. of women in analysis	No. of women with recurrence	% (95% CI)	No. of women in analysis		No. of women with recurrence	% (95% CI)	No of women in analysis	No of women with perinatal disorder	% (95% CI)	Relative Risk (95% CI)
Depression	409	115	28.12 (23.76, 32.47)	870		222	25.52 (22.62, 28.41)	6717	761	11.33 (10.57, 12.09)	2.48 (2.10, 2.94)
Anxiety	410	112	27.32 (23.00, 31.63)	871		226	25.96 (23.04, 28.86)	6713	495	7.37 (6.75, 8.00)	3.70 (3.10, 4.43)
Comorbid anxiety & depression	410	77	18.78 (15.00, 22.56)	871		154	17.68 (15.15, 20.21)	6717	303	4.51 (4.01, 5.01)	4.16 (3.31, 5.24)

### 1989-95 Cohort Participants

Participants born in 1989 to 1995 were 18 to 24 years old when completing their first survey in 2013 and 24 to 30 years during their last survey in 2018. At recruitment, they were broadly representative of other Australian women of the same age in terms of geographical distribution across Australia, marital status and age distribution (ALSWH 2019). A higher percentage of ALSWH participants held university, trade, certificate or diploma qualifications, were current smokers and physically active, though alcohol consumption was lower (ALSWH 2019).

Of 17,010 participants, 4714 (27.71%) reported a history of anxiety at baseline in 2013, compared to 18.9% of Australian women of a similar age in 2014 (ABS 2018). A total of 5870 women reported depression at baseline (34.51%), compared to 10.5% in Australian women aged 15 to 24 in 2014 (ABS 2018).

Of the 17,010 participants in the 1989-95 cohort, 728 reported experiencing depression before having a child. Of those who also provided perinatal data, 40.76% experienced a recurrence of the condition during their first perinatal period, and 40.5% during any perinatal period. This compares to a perinatal depression rate of 12.43% (*n* = 71) amongst the 571 women who did not report experiencing depression before any of their pregnancies.

Of the 618 women who reported experiencing anxiety before pregnancy, 41.48% of those who also provided perinatal mental health information experienced recurrent anxiety during their first perinatal period and 43.42% during any perinatal period. In comparison, of the 637 women who did not report anxiety before pregnancy, 18.05% (*n* = 115) experienced perinatal anxiety. In this cohort, there were 511 women who reported a history of both anxiety and depression. Nearly 45% of the women who also supplied perinatal data developed recurrent depression, 45.57% developed recurrent anxiety and 34.86% developed comorbid anxiety and depression for their first child. For any child, 45.54% of women experienced recurrent depression, 46.72% experienced recurrent anxiety and 35.37% experienced comorbid perinatal anxiety and depression. Of the women who did not report a history of both anxiety and depression in the 1973-78 cohort, 16.36% (*n* = 117) experienced perinatal depression, 18.74% (*n* = 134) experienced perinatal anxiety and 10.49% (*n* = 75) experienced comorbid perinatal anxiety and depression.

More details are provided in Tables 5 and 6.

**Table 5:** Recurrence in women from 1989-95 cohort with preconception anxiety or depression, alongside the comparison group with no such history

	Recurrence with first child			Recurrence with any child				Comparison group (at first child)				Hx vs. No Hx at first birth
Preconception disorder	No. of women in analysis	No. of women with recurrence	% (95% CI)	No. of women in analysis	No. of women with recurrence	% (95% CI)	Preconception disorder	No. of women in analysis	No. of women with perinatal depression	No. of women with perinatal anxiety	% (95% CI)	Relative Risk (95% CI)
Depression	471	192	40.76 (36.33, 45.20)	642	260	40.50 (36.7, 44.3)	**No depression**	571	71	Not calculated	12.43 (9.73, 15.14)	3.28 (2.57, 4.18)
Anxiety	405	168	41.48 (36.68, 46.28)	562	244	43.42 (39.32, 47.51)	**No anxiety**	637	Not calculated	115	18.05 (15.07, 21.04)	2.30 (1.88, 2.81)

**Table 6:** Recurrence in women from 1989-95 cohort with a history of both anxiety and depression, alongside the comparison group with no such history

	Women with history						Women with no history (at first child)			Hx vs. no Hx at first birth
	Recurrence with first child			Recurrence with any child						
Perinatal disorder	No. of women in analysis	No. of women with recurrence	% (95% CI)	No. of women in analysis	No. of women with recurrence	% (95% CI)	No. of women in analysis	No. of women with perinatal disorder	% (95% CI)	Relative Risk (95% CI)
Depression	327	146	44.65 (39.26, 50.04)	458	204	44.54 (39.99, 49.09)	715	117	16.36 (13.65, 19.08)	2.73 (2.22, 3.35)
Anxiety	327	149	45.57 (40.17, 50.96)	458	214	46.72 (42.16, 51.29)	715	134	18.74 (15.88, 21.60)	2.43 (2.00, 2.95
Comorbid anxiety & depression	327	114	34.86 (29.70, 40.03))	458	162	35.37 (30.99, 39.75)	715	75	10.49 (8.24, 12.74)	3.33 (2.56, 4.31)

### Missing data

Of the women from the 1973-78 cohort who had a child and were excluded from the denominator due not having preconception anxiety, preconception depression or preconception anxiety and depression, only 57.5 to 58.4% had complete survey data upon which to base this exclusion. For the 1989-95 cohort, this ranged from 41.0% to 47.0%. Two sensitivity analyses were performed to investigate the potential impact of this missing data.

The first used linked data from the PBS and MBS to identify additional women with a history of anxiety and/or depression for inclusion in the denominator, and recurrence rates were then recalculated. The number of additional women and their impact on recurrence rates is presented in Table 7. This process had minimal impact on the estimated proportions of recurrent anxiety or depression.

**Table 7:** Sensitivity analysis: recalculated recurrence rates from lower and upper bound analysis using linked data (%)

Cohort	Analysis	No. women added for first child	Original rate for first child	Recalculated rate for first child	No. of women added for any child	Original rate for any child	Recalculated rate for any child
1973-78	Lower bound depression	15	25.69	25.97	59	23.03	23.38
	Upper bound depression	56	25.69	26.09	113	23.03	23.63
	Upper bound anxiety	78	23.79	25.04	160	23.04	23.05
1989-95	Lower bound depression	51	40.76	41.76	78	40.50	42.64
	Upper bound depression	138	40.76	42.86	132	40.50	43.41
	Upper bound anxiety	117	41.48	42.34	128	43.42	43.62

The second sensitivity analysis randomly imputed increasing proportions of the women who had been excluded from the study as they did not report any anxiety or depression, but they also had missing survey data. A maximum of 10% was imputed as the PBS/MBS sensitivity analysis identified a proportion of less than 1% missing from the denominator. It was thus deemed unlikely the sample would be missing more than 10% of eligible women. Again, this made little difference to the estimated proportions of recurrent anxiety or depression (see Tables 8 and 9).

**Table 8:** Sensitivity analysis: recurrence proportions with varying proportions of missing data imputed (1973-78 cohort)

Percentage imputed	Proportion of RD (95% CI) First child	Proportion of RD (95% CI) Any child	Proportion of RA (95% CI) First child	Proportion of RA (95% CI) Any child
1	0.25 (0.23,0.28)	0.23 (0.21,0.25)	0.23 (0.20,0.26)	0.23 (0.20,0.25)
2	0.25 (0.22,0.27)	0.23 (0.21,0.24)	0.22 (0.19,0.25)	0.22 (0.20,0.24)
3	0.24 (0.22,0.27)	0.22 (0.21,0.24)	0.21 (0.18,0.24)	0.21 (0.19,0.24)
4	0.24 (0.21,0.26)	0.22 (0.20,0.24)	0.20 (0.17,0.23)	0.21 (0.19,0.23)
5	0.23 (0.21,0.26)	0.22 (0.20,0.24)	0.20 (0.17,0.23)	0.21 (0.19,0.23)
6	0.23 (0.21,0.25)	0.22 (0.20,0.23)	0.19 (0.16,0.22)	0.20 (0.18,0.23)
7	0.23 (0.20,0.25)	0.21 (0.20,0.23)	0.19 (0.16,0.22)	0.20 (0.18,0.22)
8	0.22 (0.20,0.25)	0.21 (0.20,0.23)	0.18 (0.16,0.21)	0.20 (0.18,0.22)
9	0.22 (0.20,0.24)	0.21 (0.19,0.23)	0.18 (0.15,0.21)	0.19 (0.18,0.22)
10	0.22 (0.20, 0.24)	0.21 (0.19, 0.23)	0.17 (0.15,0.20)	0.19 (0.17,0.21)

**Table 9:** Sensitivity analysis: recurrence proportions with varying proportions of missing data imputed (1989-95 cohort)

Percentage imputed	Proportion of RD (95% CI) First child	Proportion of RD (95% CI) Any child	Proportion of RA (95% CI) First child	Proportion of RA (95% CI) Any child
1	0.41 (0.36,0.45)	0.42 (0.38,0.46)	0.41 (0.37,0.46)	0.45 (0.41,0.49)
2	0.40 (0.36,0.45)	0.42 (0.38,0.45)	0.41 (0.36,0.46)	0.44 (0.40,0.49)
3	0.40 (0.36,0.45)	0.42 (0.38,0.45)	0.41 (0.36,0.46)	0.44 (0.40,0.48)
4	0.40 (0.36,0.45)	0.42 (0.38,0.45)	0.41 (0.36,0.45)	0.44 (0.40,0.48)
5	0.40 (0.36,0.45)	0.42 (0.38,0.45)	0.41 (0.36,0.45)	0.44 (0.40,0.48)
6	0.40 (0.36,0.45)	0.42 (0.38,0.45)	0.40 (0.36,0.45)	0.44 (0.40,0.48)
7	0.40 (0.36,0.45)	0.42 (0.38,0.45)	0.40 (0.36,0.45)	0.44 (0.40,0.48)
8	0.40 (0.36,0.45)	0.42 (0.38,0.45)	0.41 (0.36,0.45)	0.44 (0.40,0.48)
9	0.40 (0.36,0.45)	0.41 (0.38,0.45)	0.40 (0.35,0.45)	0.44 (0.40,0.48)
10	0.40 (0.36,0.45)	0.41 (0.38,0.45)	0.40 (0.35,0.45)	0.44 (0.40,0.47)

## Discussion and Conclusions

This is the first large-scale, multi-cohort study with data collected over nearly 30 years examining the perinatal relapse or recurrence of anxiety and/or depression. Consistent with smaller studies by Martini et al. (2015) and Molenaar, Brouwer et al. 2019), rates of perinatal anxiety and/or depression were higher in women with preconception anxiety and/or depression compared to women without a history of the disorders.

Women in the 1973-78 cohort with preconception anxiety experienced over three times the rate of perinatal anxiety than women who did not report the illness before pregnancy (23.04% vs. 7.11%). Similarly, the rates of perinatal depression in women with preconception depression were more than twice the rate of women without such a history (23.03% vs. 9.87%).

For participants of the 1989-95 cohort, women who reported preconception depression were found to experience over three times the rate of perinatal depression compared to women who did not (40.76% vs. 12.43%) and the rate of perinatal anxiety for women with preconception anxiety was also more than twice that of women without such a history (43.42% vs. 18.05%).

While higher than perinatal women without preconception anxiety and/or depression, the rates of perinatal anxiety and/or depression were also much higher in the 1989-95 cohort compared to the 1973-78 cohort (anxiety 43.42% vs. 23.04%, depression 40.76% vs. 23.03%). The difference in figures between the two cohorts may reflect the sharp increase in anxiety and/or depression among young women over the last decade (Duffy et al. 2019; Twenge 2020; Thapar et al. 2022; Wykes et al. 2023), perhaps a consequence of greater mental health awareness, reduced stigma, the influence of social media and/or the pressures of living in a modern, neoliberalist society (McGorry et al. 2025).

The difference in recurrence rates may also be partly due to the 1989-95 cohort being relatively young. Youth has been associated with perinatal anxiety and depression (Biaggi et al. 2016; Hutchens and Kearney 2020), and, while not the cause, early motherhood has been linked to social disadvantage, low education and unhealthy lifestyles (Lee and Gramotnev 2006), factors also associated with perinatal anxiety (Biaggi et al. 2016; Field 2018) and depression (Biaggi et al. 2016; Hutchens and Kearney 2020). Unfortunately, it was not possible to compare the two cohorts statistically, as the questions included in the surveys were slightly different between cohorts, as was how responses were categorised.

The main limitation of this study is missing data. As is common in long-term longitudinal studies, many women did not participate in every survey or fully complete each questionnaire, potentially introducing bias. Those who did participate fully in the surveys had more opportunity to report both preconception and perinatal anxiety and depression. Furthermore, it is possible that women who did not participate in a survey were unable to do so due to mental illness, or they may have chosen not to report the condition due to perceived stigma, especially around perinatal anxiety and depression. While missing data was a limitation of the study, the risk of bias was controlled for as much as possible by including two methods of sensitivity analysis, neither of which indicated any inaccuracies with the findings.

Another limitation is that participants in the 1989-95 cohort have not finished having children. They were 24 to 30 years old at their last survey, and the rates of relapse or recurrence may decrease with the addition of more women who have children later in life. To address this limitation, data from the 1973-78 cohort that encompassed the entire reproductive life course of participants was included in the analysis, however further investigation of the women from the 1989-95 cohort in another 10 to 15 years would further elucidate their rates of relapse and recurrence.

Lastly, determining whether a participant was asymptomatic of their self-reported preconception anxiety and/or depression before becoming pregnant was not possible given the retrospective nature of the data used. It was therefore impossible to discern whether experiencing perinatal anxiety and/or depression was a true relapse or recurrence, or merely a continuation of the reported illness.

Given the high rates of perinatal relapse or recurrence in women with preconception anxiety and/or depression demonstrated in this study, as well as the well-established risks to the health and development of their offspring, supporting them to remain asymptomatic during the perinatal period is a priority. These findings provide the justification and rationale for future investment in research and intervention in this area.

To facilitate any support intervention, further exploration of why some women with preconception anxiety and/or depression experience relapse or recurrence, while others do not, is required. This will guide interventions to target modifiable protective factors and determine relevant risk factors to assist in identifying women who need additional monitoring. It may also identify a high-risk group of women who are already at increased risk of perinatal anxiety and/or depression, allowing for an even more targeted intervention when resources are greatly constrained.
